# COVID-19 infection data encode a dynamic reproduction number in response to policy decisions with secondary wave implications

**DOI:** 10.1038/s41598-021-90227-1

**Published:** 2021-05-25

**Authors:** Michael A. Rowland, Todd M. Swannack, Michael L. Mayo, Matthew Parno, Matthew Farthing, Ian Dettwiller, Glover George, William England, Molly Reif, Jeffrey Cegan, Benjamin Trump, Igor Linkov, Brandon Lafferty, Todd Bridges

**Affiliations:** 1 U.S. Army Engineer Research and Development Center, Vicksburg, MS USA; 2 U.S. Army Engineer Research and Development Center, Hanover, NH USA; 3 U.S. Army Engineer Research and Development Center, Concord, MA USA

**Keywords:** Computational biology and bioinformatics, Epidemiology

## Abstract

The SARS-CoV-2 virus is responsible for the novel coronavirus disease 2019 (COVID-19), which has spread to populations throughout the continental United States. Most state and local governments have adopted some level of “social distancing” policy, but infections have continued to spread despite these efforts. Absent a vaccine, authorities have few other tools by which to mitigate further spread of the virus. This begs the question of how effective social policy really is at reducing new infections that, left alone, could potentially overwhelm the existing hospitalization capacity of many states. We developed a mathematical model that captures correlations between some state-level “social distancing” policies and infection kinetics for all U.S. states, and use it to illustrate the link between social policy decisions, disease dynamics, and an effective reproduction number that changes over time, for case studies of Massachusetts, New Jersey, and Washington states. In general, our findings indicate that the potential for second waves of infection, which result after reopening states without an increase to immunity, can be mitigated by a return of social distancing policies as soon as possible after the waves are detected.

## Introduction

The virulent SARS-CoV-2 virus is responsible for the pandemic of novel coronavirus disease 2019 (COVID-19) that began in late 2019 to ultimately afflict the global population. The first confirmed instance of COVID-19 in the United States was recorded on 21 January 2020; since then, total reported infections soared to over 4,363,511 confirmed cases and 149,375 deaths in the United States as of 29 July 2020^1,2^. By March 2020, relatively few forecasting tools were available for reliably estimating trends in reported infections^3,4^. This led to a level of uncertainty in the efficacy of social policy to control the rate of further infections in both urban and rural populations. At a federal level, the United States Army Corps of Engineers (USACE), Federal Emergency Management Agency (FEMA), and the Department of Health and Human Services (HHS) have responded to the COVID-19 pandemic with domestic relief efforts^5^, but these are primarily reactive and localized to existing outbreak hotspots. Limitations in the availability of personal protective equipment (e.g., N95 masks) and critical care tools (e.g., ventilators) require a more proactive approach to understanding where and when the most severe outbreaks will occur. The largely unknown efficacy of social policy to reduce the interactions between infected and susceptible individuals that lead to new COVID-19 infections makes a proactive approach especially important. Even more uncertain is the potential for sequestered yet susceptible individuals to fuel a “second wave” of infections. Therefore, we can expect that the timing of social distancing policies will correlate with future infection waves through population immunity, other dynamic features of population movement, and accessibility to infected individuals.

A link between social distancing and infection dynamics has been noted before in studies of other epidemics. For example, Herrera-Valdez et al.^6^ analyzed infection data from the 2009 Mexican epidemic of swine-origin influenza virus A/H1N1 (H1N1pdm), finding that transport of individuals between states was insufficient by itself to explain the infection dynamics; only when social distancing was adjusted via changes to a transmissibility parameter was the model able to fit all three waves of A/H1N1 infections across Mexico. Throughout 2020, the US Centers for Disease Control (CDC) issued restrictions on both domestic and international air travel^7^, and many states issued individual restrictions. Although there are yet no comprehensive retrospective analyses that reveal the impact of travel restrictions on the development of the domestic COVID-19 epidemic, at least some initial studies^8^ suggest travel restrictions primarily act to delay geographical COVID-19 progression. To drive an epidemic to extinction may therefore require additional mitigation measures, such as social distancing^9^.

Here we report on efforts to deduce how statewide policies meant to affect population mobility and disease transmissibility may impact US COVID-19 progression by analyzing infection data reported by the health departments of Massachusetts, New Jersey and Washington—states with extensive, early COVID-19 outbreaks. The traditional Susceptible-Exposed-Infected-Recovered (SEIR) modeling framework cannot, as currently formulated, capture the effects of dynamic variation of individual level social behavior at population scales, because the conditional statistics that model these interactions are fixed in time. We address this problem by developing new methods that allow a SEIR framework to accommodate dynamic trends in a diurnally updated time series, implemented in a tool we refer to as the Engineer Research & Development Center (ERDC) SEIR model.

## Methods

### Data acquisition

Available COVID-19 datasets primarily represent data aggregated from the individual reports of state health departments and are made publicly available by several organizations, most notably by the Johns Hopkins University (JHU)^1^ and USAFacts (https://usafacts.org/visualizations/coronavirus-covid-19-spread-map/). We chose to use the USAFacts datasets that report COVID-19 cases for all 3141 counties and parishes of the United States, whereas the JHU datasets report a mixture of municipalities and multiple scales (state and county) in addition to a selection of foreign locations. The USAFacts datasets represent the cumulative number of positive COVID-19 cases reported over time. As the pandemic proceeds across the United States, we should therefore expect these data to represent trends that rise exponentially with the first COVID-19 reports and to plateau when the infection wanes. The inflection point of this cumulative reported infection curve therefore represents the peak in new daily reported infections.

There are several unique challenges associated with modeling COVID-19 infection data. First, datasets are continually updated to include new reports that serve to reduce the uncertainty in infectious trends as the disease progresses with time. Second, there are few studies informing key epidemiological parameters that help to model progression of COVID-19 in susceptible populations, leading to greater uncertainty surrounding values for the latency period (3–10 days), *Z*, or the duration of symptoms (5–14 days), *D*^9–11^. Other challenges exist, such as widespread testing delays that potentially contribute to a sampling bias in the available data^12^. SARS-CoV-2 is an RNA virus, meaning that reverse transcription polymerase chain reaction (RT-PCR) testing is required to detect the presence of virus in samples^13^. The RT-PCR testing is often slow and testing locations have limited capacity to process existing samples and adapt to increased demand. Finally, a combination of variation in disease onset and severity, and testing limitations leads to an effective delay in the number of new positive cases reported by health agencies. The number of COVID-19 cases reported today therefore represents only a fraction of the total number of infections at some time in the past. Timescales of disease progression compound directly with observational delays to obscure the current magnitude of the health crisis as seen through reported COVID-19 cases. To leverage these data for understanding COVID-19 infection dynamics, we must first account for the mechanisms associated with infection and disease progression, followed by an accounting of how infected individuals are tested and reported to authorities.

### Model formulation

For a population in which the total number of individuals remain fixed over time, mathematically-speaking, we assign each individual to any one of four possible disease states: susceptible, exposed, infected, and recovered. These states are consistent with standard epidemiological descriptions of those who are susceptible to COVID-19 infections, or those exposed to infected individuals through either direct (e.g., airborne particulates) or indirect interactions (e.g., interaction with common infected surfaces). Individuals in such an exposed state will develop a symptomatic infection after a latency period. Finally, we assume that infected individuals will transition into a recovered state in which individuals are no longer contagious. While those in the recovered state include fatalities and non-lethal recoveries, this could also include those who are sick in the hospital but isolated and unable to spread the disease. To remain consistent with the available data, we make a distinction between individuals in an infected state that have had a positive test reported to the relevant health department and those that remain unreported. Other models have also distinguished between infected and infected-reported COVID-19 infections^14^. This allows the model to capture individuals who have been tested and have a higher transmission rate historically due to early screening qualifications presented by the CDC to qualify for testing, including physical symptoms, recent travel to an outbreak site, and direct contact with someone with a known infection^15^.

We make a number of additional assumptions about each modeled population to further simply our approach. First, we assume that the SARS-CoV-2 virus affects all individuals identically, which is unrealistic because it ignores a potentially significant source of variation within the population. For example, there is substantial clinical evidence that COVID-19 symptoms are more severe in older individuals than younger ones^16^. Although older individuals may be overrepresented in official fatality estimates, we are not presently aware of any evidence related to an age dependent bias in exposure potential to COVID-19, which would affect our interaction model between susceptible and infected individuals. Otherwise, differences between spatially separated populations will manifest in our model through different parameter values, such as the disease transmissibility (see below). For single population centers, our assumption of homogeneity is more appropriate for larger population centers, wherein the coefficient of variation is smaller than in population centers with fewer individuals. This makes a deterministic description appropriate for modeling larger geographic scales of interest. If these assumptions hold, then we may reasonably ignore the statistics of transition between the four disease states described above. We assume that state transitions proceed from susceptible to exposed to infected, and, finally, to the recovered state, at which point recovered individuals cannot be re-infected and are no longer infectious. These assumptions lead to a set of coupled, deterministic ordinary differential equations that together model the number of individuals in each disease state over time. State-transition statistics are therefore replaced by a number of “currents,” each of which describe either the number of individuals entering or leaving their respective state per unit time, and the balance of these currents defines a rate of accumulation or depletion of individuals associated with a disease state over time. The mathematical details of the model are available in the Supplementary Methods.

We model these deterministic transition rates using a number of parameters with common epidemiological interpretation. In addition to the latency and duration mentioned above, we include the disease transmissibility, $\beta$, which is the potential per unit time that an interaction with an infectious individual develops into an infection, the fraction of infected individuals that seek out and receive a RT-PCR test, *α*, and a weighting factor, *µ*, which attenuates the transmissibility of individuals with an unreported infection. These parameters are sufficient to estimate the effective reproduction number, *R*_*e*_, which accounts for the number of additional infections that originate from each infected individual.

## Results

### Model segmentation captures dynamical trends in COVID-19 datasets

Shelter-in-place orders (SIPOs) are premised on a hypothesis that if COVID-19 spreads primarily through interactions between infected and susceptible individuals, then reducing the number of these interactions will reduce the number of individuals that develop an infection. By 20 April 2020, at least 40 state governors had issued SIPOs, and a statistical analysis of data acquired for a 3-week period between 8 March and 17 April linked these policy decisions to a 44% reduction in the number of cumulative infections that followed policy implementation^17^. Thus, SIPO compliance appears to correlate with a reduction in COVID-19 cases, possibly through a reduction in population mobility. This prompts the question of whether a population mobility mechanism can be used to link dynamical features in COVID-19 time series data with SIPO dates.

We cannot directly incorporate a reduction of population mobility into our compartmentalized model because our assumption that accumulation or depletion rates are “reaction limited” eliminates their explicit spatial dependence. In our model, new COVID-19 infections emerge from interactions between susceptible and infected individuals, so we could achieve the effects of a reduced population mobility by altering the accessibility of susceptible individuals to infected ones. As shown in the Supplementary Material, at a date in the time series associated with a SIPO, we partition a fraction, $\gamma$, of the susceptible population at time *t*, *S*_*t*_, into one or more subsets, $\left(1-\gamma \right){S}_{t}$, which remain inaccessible to infected individuals for the duration of the SIPO period. We note that this methodology lacks an associated period of compliance, which is at least as long as the COVID-19 latency^17^.

Although we can directly manipulate the total number of potential exposure interactions by sequestering some of the susceptible population, this is a fundamentally different mechanism from the type of social distancing behavior studied by authors that fine-tune the transmissibility, $\beta$, which accounts for the conditional probability that an interaction will lead to exposure, and, ultimately, to an infection. However, this transmissibility parameter is not specific enough to distinguish between the types of distancing behavior that may play a significant role in reducing or eliminating the spread of a disease. For example, some studies indicate that voluntary social distancing, which refers to distancing behaviors absent official guidance, may factor more strongly into early epidemics than when state-level policies become established^18^, and that distancing effectiveness could ultimately depend on the perceived risks of infection^19^.

Our model makes a rather strong assumption that $\beta$ remains constant across the whole of the period of analysis; in other words, we assume that any social distancing associated with individualized decisions to increase the physical distance of personal contact is persistent and equal in effectiveness across the analysis period. Nevertheless, this assumption allows us to study how variation in population mobility affects COVID-19 progression free of the confounding interactions associated with more complex and nuanced proximity-based interactions. One downside of this approach is that it cannot detect how more complex “second order” social behaviors affect epidemiological progression. For example, a population may change its distancing behavior given a change in public awareness of the disease progression or if the public health incentives change over time^20^. Thus, we could hypothesize that $\beta (t)$ change over a timescale that captures the extent of mobility-related social feedback. Some recent work has sought to filter these dynamics from the available data^21^, finding that $\beta (t)$ may change more slowly over months near the start of the COVID-19 pandemic (February to April), followed by a period of more rapid change at the scale of weeks (April to May and beyond). In the period before a state-wide SIPO, this transmission timescale correlates with mobility-related metrics that account for travel to work, grocery shopping, retail shopping (including restaurants), and other places^22^, suggesting that $\beta (t)$ is not entirely determined by the number of proximal interactions (i.e., a rate-limited disease production), but might also be affected by the location of these interactions (i.e., a transport-limited disease production). These effects are further obscured by uncertainty between the active and latent prevalence of confirmed cases of COVID-19^23^. In view of these considerations, additional analyses methods will be needed to extract a dynamic profile for $\beta (t)$ that remains statistically consistent with the available data, yet robust to the inherent uncertainties associated with aggregating disparate mobility and public health datasets.

We fit our deterministic model to new daily infection data using a Bayesian approach, which requires that a probability distribution be defined over the model parameters conditioned on daily new infection data. This distribution depends on the nonlinear form of our model and does not follow any standard form. Computationally, we employ a combination of maximum a posteriori (MAP) estimation and Markov chain Monte Carlo (MCMC) sampling. Our approach first identifies a set of model parameters that maximize the likelihood of observing the reported COVID-19 time series data and then explores the entire posterior distribution to characterize the uncertainty in the model parameters. Posterior samples of the model parameters get propagated through the model equations to characterize the model’s predictive uncertainty. This Bayesian approach requires the definition of a prior probability distribution for the model variables and a statistical error model for the difference between predictions and observations. We adapt previously reported prior distributions for the model parameters^14^, while using a combination of log-normal and uniform distributions to represent our prior knowledge of the initial conditions of the modeled subpopulations. We then make the common assumption that differences at time *t* between model predictions and data are normally distributed, with a constant variance that is estimated in a hierarchical Bayesian formulation alongside the model parameters.

**Figure 1:**
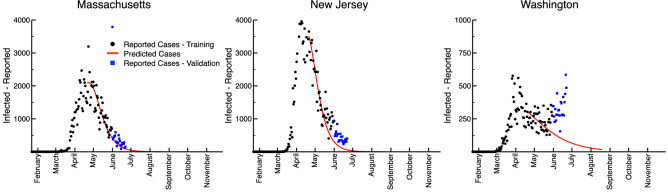
The ERDC SEIR model forecasts for Massachusetts, New Jersey, and Washington. The black dots represent the number of active cases as represented by data, while the red line is the median number of active cases predicted by the model.

We focus our model optimization on the last 28-day segment of time-series infection datasets for each of the three states we examined. As human behavior can be dynamic, the model fit to these truncated data provide a picture of the present disposition of a state’s residents in relation to SIPOs. This can be seen in our fits for Massachusetts, New Jersey, and Washington, in which the deterministic model was fit to time-series data from 22 January 2020 to 31 May 2020 (Fig. 1). Here, black circles illustrate the new daily reported infections, whereas the red curves illustrate the forecast associated with the median of the ensemble trajectories identified through our Bayesian curve-fitting method. Finally, the blue squares illustrate the daily reported infections since the model’s training, from 01 June 2020 to 22 June 2020. Massachusetts and New Jersey have tracked well with the model’s predictions, while Washington has seen a positive trend in new cases since the beginning of June not predicted by the model. This suggests either a change in SIPOs policies in Washington, such as the introduction of the phased returns that began 01 June 2020^24^, or changes in adherence and population behavior, resulting in outbreaks such as those in agricultural businesses and long-term care facilities such as those seen in eastern Washington^25^, driving the increase. This highlights the primary limitation of the model: the resulting forecasts are only applicable given a continuance of SIPOs policy and social behaviors.

### Linking policy decisions to additional infection waves

Model fits to new daily case data are greatly improved if we sequester a fraction of susceptible individuals to limit access to infected ones (Fig. 1), but clearly these individuals do not indefinitely remain immobile. If a goal of SIPOs is to minimize the extent and severity of COVID-19 infections, then it seems reasonable to lift them once infections fall below an acceptable level. What is this level and what happens if SIPOs are removed too early or ignored, as we have seen in Washington and other states?

The Spanish Flu pandemic of 1918–1919 offers some insight, specifically in that it presented as three successive waves of infections across the world^26,27^. Mathematical modeling of this disease at a population level suggests that its three infectious waves were rooted in the time dependence of its population-averaged transmission rate^28^. Social factors, such as changes in population mobility, in addition to others, can facilitate these trends. However, the Spanish Flu pandemic is thought to have been affected by antigenic drift, which could also contribute changing immunity and transmission rates^29,30^. A more general survey of mechanisms that might reproduce multiple infection waves for influenza pandemics suggests that a time-dependent transmission rate is sufficient to fit long-term infection trends with low error^31^. This study also suggested that only very strong intervention (i.e., approx. 90% reduction in initial susceptibility) eliminated the appearance of second waves. Although the molecular details of these viruses differ, these studies suggest that concepts such as a time-dependent transmissibility and manipulation of susceptible populations are general enough to describe how multiple waves of COVID-19 infections could evolve in the future.

**Figure 2:**
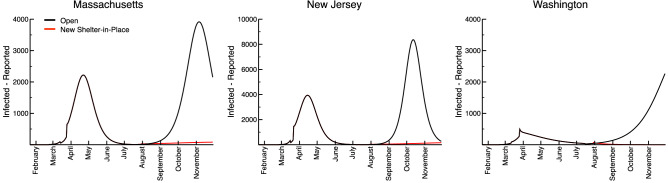
The ERDC SEIR second wave hypothetical scenarios for Massachusetts, New Jersey, and Washington, assuming that SIPOs are lifted on 15 July 2020. The black curves represent the median number of reported infections predicted by the model if no population immobilization policies (e.g., shelter-in-place orders) are put in place to mitigate the second wave. The red curves represent the median number of reported infections predicted by the model if shelter-in-place policies are enacted 2 weeks beyond 15 July.

We demonstrate a hypothetical second wave scenario for Massachusetts, New Jersey, and Washington in Fig. 2. A fraction of the population (70%, in this example) is reserved in isolation early in the simulation, then released on 15 July 2020 when the SIPOs are relaxed in these scenarios. We then forecast through the end of November for each state. States then see the peak of the second wave in mid-October to mid-November 2020. Note that, without the reimplementation of social distancing, the second waves are more severe than the first, reaching over twice the number of active cases in the peak days compared to the first wave (Fig. 2, black). We also test the scenario in which states stand up SIPOs starting 2 weeks into the second wave, upheld through the end of the simulation. These policies result in a nearly-complete attenuation of the second wave (Fig. 2, red). This result highlights the necessity to analyze current policies and prepare populations to socially distance themselves as quickly as possible once another wave is detected.

The effective reproductive number, R_e_, is a metric of the transmissibility of the virus; viruses with R_e_ greater than 1 are likely to spread while those with R_0_ less than 1 are likely to die out and become extinct. An early review of R_e_ estimates for China found the average to be 3.28, with a median of 2.79^32^. We have implemented the next-generation matrix (NGM) method to estimate the R_0_ for each day^14,33^. This allows us to dynamically track and estimate the R_e_ and determine how changes in policy affect the transmissibility of the virus in state level populations.

**Figure 3:**
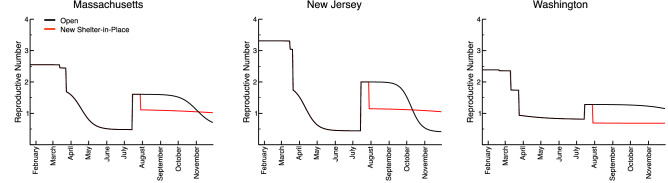
The dynamic reproductive number for SARS-CoV-2 over the two wave scenarios for Massachusetts, New Jersey, and Washington as presented in Fig. 2. The black curves represent the reproductive numbers over time for the open scenario, while the red curves represent the reproductive numbers over time for the shelter-in-place scenarios.

We demonstrate in Fig. 3 the daily changes in the R_e_ for the completely open and sheltered-in-place second wave scenarios for the three states. Initially, the R_e_ ranges between 2–3.5 before dropping due to changes in social distancing policy and behavior in the model. These then gradually decrease until R_e_ is less than 1. If current conditions are maintained, R_e_ = 1 quantifies a threshold below which the population will eventually return to a disease-free state. Once the SIPO is lifted on 15 July 2020, the R_e_ increases back to approximately 1–2 due to an influx of people from isolation, peaking in advance of secondary infection waves. These R_e_ values fall to below 1 again in the “open state” scenario, trending monotonically down throughout the extent of the second wave. However, reimplementation of the SIPO 2 weeks after the second wave begins, sees R_e_ values fall back below 1 where they persist, hearkening back to the attenuation of the curve we demonstrated in Fig. 2. This suggests that observation of a dynamic R_e_ metric could be used to monitor current and future waves of infections over moderate predictive timescales, and to justify changes in state policy and social behavior to mitigate public health impacts.

## Conclusion

Ultimately, the ERDC SEIR represents a powerful tool to monitor the COVID-19 pandemic, as well as future outbreaks of COVID-19 and other diseases by accounting for a range of drivers and stressors related to population behavior and policy activity as well as daily-adjusted parameters to calculate conditions that may generate future waves of pandemic activity. Our current forecasts are published as part of the COVID-19 Forecast Hub, with data available at https://github.com/erdc-cv19/covid19-forecast-hub/tree/master/data-processed/USACE-ERDC_SEIR. In the near future, we anticipate making second wave forecasts publicly available to further support USACE and FEMA responses and help guide state and federal policy, while maintaining the production and distribution of forecasts. Finally, sudden events that bring people together or induce mobility, such as political protests and rallies or potential evacuations resulting from hurricanes during the 2020 storm season have the potential to complicate our ability to manage the COVID-19 pandemic, especially if people carry the virus into large congregations of people, shelters or even other cities as well as emergency response teams being exposed. Overall, the ability to understand the conditions and activities that fuel future waves can give policymakers and emergency responders valuable time to prepare for and absorb COVID-19 disruptions, as well as consider actions that may future mitigate disease spread.

Opinions, interpretations, conclusions, and recommendations are those of the authors and are not necessarily endorsed by the U.S. Army. The authors do not claim any conflicts of interest.

## Supplementary Information

Supplementary Information.
