# Supplementary material for: COVID-19 infection data encode a dynamic reproduction number in response to policy decisions with secondary wave implications
Source: Sci Rep. 2021 May 25;11:10875. doi: 10.1038/s41598-021-90227-1 (PMC8149655; doi:10.1038/s41598-021-90227-1)
Supplement: Supplementary file 1 — Supplementary Information. [file 41598_2021_90227_MOESM1_ESM.docx]

**COVID-19 infection data encode a dynamic reproduction number in response to policy decisions with secondary wave implications**

**Supplemental Material**

Michael A. Rowland, Todd M. Swannack, Michael L. Mayo, Matthew Parno, Matthew Farthing, Ian Dettwiller, Glover George, William England, Molly Reif, Jeffrey Cegan, Benjamin Trump, Igor Linkov, Brandon Lafferty, Todd Bridges

**Model overview**

The ERDC SEIR model simulates the number of COVID-19 infections in a state or metropolitan statistical area (MSA). The model is a modification of classic SEIR models that distinguish between reported and unreported cases, similar to an approach employed by a team from Columbia University (Li et al, 2020). The ERDC SEIR model however, also employees another compartment to represent people that are isolated from larger population and will not be exposed to the virus. The model parameters are fit independently for each state and metropolitan area to match time-series data of confirmed infection reports. This calibration process is repeated daily as new data becomes available and is performed on ERDC’s high performance computing cluster. More details are provided below.

| 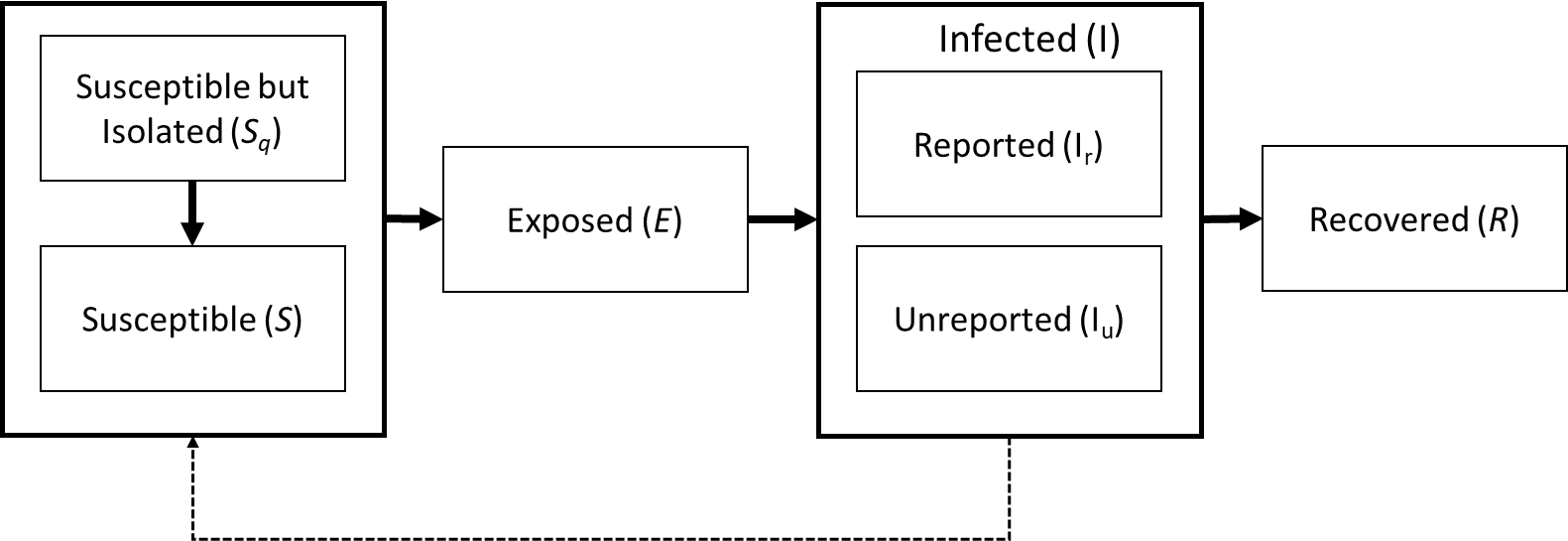 |
| --- |
| **Figure S1.** Conceptual model of the disease states. Healthy individuals are exposed to COVID-19 through infected individuals, and only a fraction of symptomatic individuals receive a test. All infected individuals “recover,” which we use to account for those who become immune from further infection or die from the symptoms. The dotted line indicates a correlation between the number susceptible individuals and those infected. |

**Model formulation**

The progression of the disease by the transition of individuals in a population through 5 states, as illustrated in Figure S1. These states are: Susceptible (S), Exposed (E), Infected (I) or Recovered/Removed (R). The model makes a number of assumptions. First, it assumes that all individuals of a population can be treated identically; that is, there is no population variation in the transition rates between infection states. Another assumption of this type of model is that individuals are “well mixed,” by which we assume that rates of disease progression do not depend upon the positions of any individuals within the subpopulations. Therefore, we do not account for any spatial heterogeneity in population density (e.g., cities vs. rural), which would otherwise directly affect the frequency of individual interactions that drive disease spread. Another important assumption we make is that populations are large enough that fluctuations driven by some of the individual variation in disease progression are small and can be ignored, which is common in population and epidemiological modeling. As a consequence, the individuals move between disease subpopulations (e.g., from susceptible to exposed) at deterministic rates. Finally, we have imposed a constraint in which individuals cannot return to a previous disease state, which reflects our presumption that those who recover are immune from further infection. Despite these various approximations and simplifying assumptions, the model is flexible enough for adaption to future scenarios. For example, population density can be accounted for using this conceptual approach by creating additional compartments to describe subpopulations at a scale under which further variation is ignored. However, such changes come at a cost of introducing additional model parameters, which is typically undesirable due to concerns of overfitting and non-uniqueness.

More specifically, these assumptions lead to four ordinary differential equations (ODEs), each of which define evolution of the four disease states:


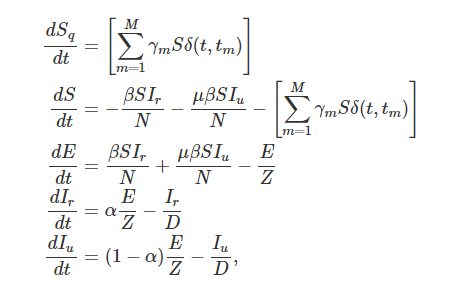


Collectively, these deterministic equations describe the evolution of the number of individuals susceptible to the disease, $S$, exposed to the disease after contact with infected individuals, $E$, those individuals with reported/tested infections, $I_{r}$, and individuals with unreported infections, $I_{u}$. The overall number of ACTIVE cases being tracked by authorities each day is given by $I_{r}(t-T_{delay})$; i.e., the number of infected individuals being tracked by $I_{r}$ is shifted deterministically by an amount equal to the delay time, $T_{delay}$. The current model is entirely deterministic, which may be stochastically adjusted in the future to account for uncertainties in the values for some of the fitted parameters (e.g., number of exposed individuals at $t=0$, which is not measured).

**Model Calibration and Uncertainty Quantification**

There are 9 unknown quantities in the above model: the initial conditions ${[S}_{0}, E_{0}, I_{r0}, I_{u0}]$ and the parameters $[\alpha,\beta,\mu,Z,D]$. To characterize these 9 quantities, we use daily observations of the number of active cases, denoted here by ${\{d}_{1}, d_{2}, ...,d_{T}\}$, where $T$ is the total number of days with observations. These observations are related to the value of reported infections $I_{r}(t)$ in the model equations. We employ a Bayesian formulation of this model calibration problem and define a posterior probability distribution over the 9 model variables given the observations. Maximizing the density of this posterior distribution is akin to nonlinear least squares and results in a single point estimate of the most likely model variables. These parameters are called the Maximum aposteriori (MAP) point. The posterior distribution however, also contains information about uncertainty in parameters that can result from observation noise or ill-posedness, meaning in this context that multiple parameter settings can match the data equally well. The posterior distribution depends on the nonlinear model, and does not therefore fall into a canonical family of distributions that can be sampled directly (e.g., Gaussian). We therefore employ a simple random walk Metropolis Markov chain Monte Carlo (MCMC) algorithm with a proposal based on the Laplace approximation of the posterior density at the MAP point to generate samples of the posterior. These samples are then propagated through the model equations to characterize uncertainty in our predictions.

Defining the posterior distribution requires us to define two things: (1) a prior probability distribution over the model variables that describes knowledge we may have about the variables before observing the data (e.g., known bounds, positivity) and (2) a statistical error model for the difference between the model predictions and observations. For the parameters $[\alpha,\beta,\mu,Z,D]$ we adapt the prior distribution used in the Columbia model. For the initial conditions ${[S}_{0}, E_{0}, I_{r0}, I_{u0}]$ we employ a combination of log-normal and uniform distributions to represent our prior knowledge. Notice that even though the prior distribution is constructed from canonical distribution families, the posterior will not be of a standard form because it depends on the model equations. For the statistical error model, we make a common assumption that at any time $t_{i}$, the errors between the model predictions and observations, ${e_{i} = I}_{r}(t_{i})-d_{i}$, are normally distributed with some constant variance $\sigma^{2}$. This variance accounts for both observation noise and the difference between our model and reality. We employ a hierarchical formulation and estimate $\sigma^{2}$ along with the model variables. An inverse-Gamma hyper prior is used.
